# Circulating Cellular Adhesion Molecules and Cognitive Function: The Coronary Artery Risk Development in Young Adults Study

**DOI:** 10.3389/fcvm.2017.00037

**Published:** 2017-05-24

**Authors:** Cynthia Yursun Yoon, Lyn M. Steffen, Myron D. Gross, Lenore J. Launer, Andrew Odegaard, Alexander Reiner, Otto Sanchez, Kristine Yaffe, Stephen Sidney, David R. Jacobs

**Affiliations:** ^1^Division of Epidemiology and Community Heath, University of Minnesota, Minneapolis, MN, USA; ^2^Department of Laboratory Medicine and Pathology, University of Minnesota, Minneapolis, MN, USA; ^3^Laboratory of Epidemiology, Demography, and Biometry, National Institute on Aging, Bethesda, MD, USA; ^4^Department of Epidemiology, University of California, Irvine, CA, USA; ^5^Department of Epidemiology, University of Washington, Seattle, WA, USA; ^6^Division of Renal Diseases and Hypertension, University of Minnesota, Minneapolis, MN, USA; ^7^Department of Psychiatry, University of California, San Francisco, CA, USA; ^8^Division of Research, Kaiser Permanente Oakland, Oakland, CA, USA

**Keywords:** cellular adhesion molecules, intercellular adhesion molecule-1, vascular cellular adhesion molecule-1, selectin, cognitive function, Rey Auditory Verbal Learning Test, Digit Symbol Substitution Test, Stroop Test

## Abstract

**Objective:**

Higher circulating concentrations of cellular adhesion molecules (CAMs) can be used as markers of endothelial dysfunction. Given that the brain is highly vascularized, we assessed whether endothelial function is associated with cognitive performance.

**Method:**

Within the Coronary Artery Risk Development in Young Adults (CARDIA) Study, excluding *N* = 54 with stroke before year 25, we studied CAMs among *N* = 2,690 black and white men and women in CARDIA year 7 (1992–1993, ages 25–37) and *N* = 2,848 in CARDIA year 15 (2000–2001, ages 33–45). We included subjects with levels of circulating soluble CAMs measured in year 7 or 15 and cognitive function testing in year 25 (2010–2011, ages 43–55). Using multiple regression analysis, we evaluated the association between CAMs and year 25 cognitive test scores: Rey Auditory Verbal Learning Test (RAVLT, memory), Digit Symbol Substitution Test (DSST, speed of processing), and the Stroop Test (executive function).

**Result:**

All CAM concentrations were greater in year 15 vs. year 7. Adjusting for age, race, sex, education, smoking, alcohol, diet, physical activity, participants in the fourth vs. the first quartile of CARDIA year 7 of circulating intercellular adhesion molecule-1 (ICAM-1) scored worse on RAVLT, DSST, and Stroop Test (*p* ≤ 0.05) in CARDIA year 25. Other CAMs showed little association with cognitive test scores. Findings were similar for ICAM-1 assessed at year 15. Adjustment for possibly mediating physical factors attenuated the findings.

**Conclusion:**

Higher circulating ICAM-1 at average ages 32 and 40 was associated with lower cognitive skills at average age 50. The study is consistent with the hypothesis that endothelial dysfunction is associated with worse short-term memory, speed of processing, and executive function.

## Introduction

Decline in cognitive function is a concern among older adults as it rises with age (1, 2) and leads to loss of independent function (3). Preserving cognitive function among the non-demented general population remains an important public health challenge. Because the brain is highly vascularized, impaired endothelial function may play a role in progression to dementia.

Endothelial damage is considered to be expressed by elevated levels of cellular adhesion molecules (CAMs) situated on vascular endothelial cells. Adhesion molecules expressed on endothelial cells are linked with atherogenesis (4). Additionally, endothelial dysfunction, expressed by greater CAMs reduces vasodilation and correlates with microvascular impaired endothelium dependent vasodilation (4). More specifically, E-selectin and P-selectin mediate the initial rolling of leukocytes while vascular cellular adhesion molecule-1 (VCAM-1) and intercellular adhesion molecule-1 (ICAM-1) firmly attach and participate in migration of leukocytes (5). The chemokine fractalkine expresses on endothelial cells and plays an important role in leukocyte adhesion and migration (6). With endothelial dysfunction, the capacity to maintain homeostasis reduces, predisposing to vascular disease and brain hypoperfusion (7–9). However, studies published so far have not been able to establish the temporality between endothelial dysfunction and neurodegenerative disease. Even less attention has been given to the relationship between endothelial dysfunction and lower cognitive function among healthy adults. Therefore, it would be ideal to collect measurements of endothelial dysfunction biomarkers before symptoms of neurodegenerative diseases occur in a population-based study or signs and symptoms of cognitive dysfunction occur among healthy adults.

The objective of this study was to assess the association between endothelial function and cognitive test scores. In the Coronary Artery Risk Development in Young Adults (CARDIA) Study, endothelial dysfunction was measured by circulating CAMs, namely sICAM-1, VCAM-1, P-selectin, E-selectin, and fractalkine in calendar years 1992–1993 and 2000–2001, whereas cognitive test scores were obtained during 2010–2011. We hypothesized that higher concentrations of CAMs were associated with worse cognitive test scores. Worse cognitive test scores in middle age are of interest because they are plausibly on the trajectory to cognitive impairment. If higher circulating CAMs, indicating greater endothelial dysfunction, are associated with worse cognitive test scores measured 18 years later in young middle-aged adults, it may indicate that young adults with worse endothelial function are on a risk trajectory for developing cognitive impairment in later life. In our study, we conceptualized that poor vascular health, indicated by increased plasma level of CAMs could adversely affect cognitive skills, including worse short-term memory, problem-solving, and executive function (10–12).

## Materials and Methods

### Subjects

#### Study Sample

Coronary Artery Risk Development in Young Adults is a prospective cohort in four-field centers: Birmingham, AL, Chicago, IL, Minneapolis, MN, and Oakland, CA. The study included 5,115 black and white, men and women aged 18–30 years at baseline in 1985 and 1986 (13). Institutional Review Board approval and signed, and written informed consent were obtained from all participants at each study center. For this study, participants examined in year 7 and/or 15 who performed cognitive testing at year 25 were eligible. A flowchart of inclusions and exclusions is provided as Figure S1 in Supplementary Material. Excluded from the analysis were those who did not attend in year 7 or 15 (*n* = 1,029 in year 7 analyses and *n* = 1,443 in year 15 analyses), one participant who dropped out, stroke/transient ischemic attack before year 25 (*n* = 54), no CAMs measured (*n* = 1,172 in year 7, *n* = 816 in year 15), no cognitive function tests in year 25 (2010–2011) (*n* = 1,759), and those who missed covariates (*n* = 1,523 at year 7 and *n* = 1,588 at year 15). Therefore, we included up to 2,690 participants in any analysis involving year 7 CAMs (1,798 for ICAM-1; 2,655 for VCAM-1; 2,662 for P-selectin; 2,083 for E-selectin; 2,468 for fractalkine) and up to 2,848 participants in any analysis involving year 15 CAMs (2,665 for ICAM-1; 2,775 for VCAM-1; 2,788 for P-selectin; 2,645 for E-selectin; 2,578 for fractalkine). The number of participants varied by CAM because not all samples were made available to the authors by the CARDIA leadership. Furthermore, concerning ICAM-1, the assay in some participants was invalid because we lacked a relevant single nucleotide polymorphism (SNP). Therefore, the number of participants with assayed ICAM-1 was substantially lower compared to other CAMs in year 7. All assayed values entered our analysis; we did not exclude any people with extremely low or high values.

### Measurements

#### Blood Collection and Measurements of CAMs

Blood samples at CARDIA years 7 and 15 were processed and stored at −70°C until blood samples were shipped on dry ice to the central laboratory. Participants were asked to fast for at least 12 h, avoid excessive physical activity, and abstain from smoking for at least 2 h before being examined. Circulating soluble CAMs were assayed at the Molecular Epidemiology and Biomarker Research Laboratory in the University of Minnesota as part of two ancillary studies: the Young Adult Longitudinal Trends in Antioxidants and a grant from the American Recovery and Reinvestment Act of 2009.

All CAMs were assayed with sandwich ELISA methods from R&D systems (Minneapolis, MN, USA) (14). Serum ICAM-1 from years 7 and 15 exams were diluted 10- and 400-fold, and plasma VCAM-1 samples were diluted 21-fold. The within plus between day coefficients of variation (CV) were <10% for ICAM-1, 8.4% for VCAM-1, 10.5% for P-selectin, 7.7% for e-Selectin, and 7.5% for fractalkine. Because assays were performed several years apart (year 15 ICAM-1 and P-selectin in 2003, the remaining variables in 2012), we recalibrated year 7 sICAM-1 and P-selectin for compatibility with the early assays as previously described (14). We note that the assay depends on the rs5491 SNP in the ICAM-1 gene, almost solely restricted to black participants, which alters the ICAM-1 protein and biochemical detectability. While we had this polymorphism information in most participants, we lacked it in 223 persons at year 7, so their ICAM-1 assays were not usable. In people with missing year 15 CAM, we substituted the recalibrated year 7 value. Specifically, the year 7 CAM value was recalibrated according to the simple linear regression of the year 15 CAM on the year 7 CAM among pairs where both were assayed.

#### Measurements of Cognitive Function

Cognitive function tests including Rey Auditory Verbal Learning Test (RAVLT), Digit Symbol Substitution Test (DSST), and Stroop Test were administered in year 25 (2010–2011). The RAVLT examined the verbal learning and memory by assessing the ability to correctly memorize and recall 15 words (15). RAVLT used in CARDIA consists of five presentations of the word list with recall, one presentation of a 15-item interference list with recall, and a short delay free recall of the list. Approximately 10 min later, participants were asked to recall the original 15-item word list, unaided. The number of words correctly recalled after this long delay was used in the analyses. A greater number of words recalled (range 0–15) indicated better cognitive performance.

The DSST measured psychomotor speed, sustained attention, and working memory (16). DSST was carried out by asking participants to fill in the empty boxes, substituting the correct corresponding symbol. A greater number of digits correctly substituted (range 0–133) indicated better cognitive performance.

The Stroop Test evaluated executive function by assessing the ability to view a complex visual stimulus and to respond while suppressing the responses to another dimension (17). The test was scored by the time to correctly state the ink color of the words plus number of errors in a minute; thus, a higher score indicated worse cognitive performance.

#### Other Measurements

Standard structured interview or self-administered questionnaires were used to obtain demographic and behavioral information among CARDIA participants at each examination. Educational status was quantified as the self-reported number of years of schooling completed. Smoking status was self-reported and classified as never, former, or current smokers. A physical activity score was derived from the CARDIA physical activity history. Alcohol intake was quantified from an alcohol use questionnaire. Body weight was measured using a balance beam scale and height (without shoes) using a centimeter ruler. Body mass index (BMI) was calculated as weight (kg)/height (m^2^) (18). Plasma lipids were measured enzymatically by Northwest Lipids Research Laboratory (Seattle, WA, USA). Plasma C-reactive protein was measured using a BNII nephelometer (Dade Behring, Deerfield, IL, USA), with a CV 2.3–4.4% and inter-assay CV 2.1–5.7% (19).

Diabetes was determined by either being diagnosed with fasting glucose level ≥126 mg/dL, self-report of antidiabetic medication, or a 2-h postload glucose ≥200 mg/dL (20). Elevated blood pressure was determined using the cut points 130 mmHg (systolic blood pressure) and 85 mmHg (diastolic blood pressure) or taking antihypertensive medication. We adjusted for the year 7 measurement of the *a priori* diet quality score, a diet pattern that has been previously described and was previously shown to correlate with CAMS (14) and cognitive function (21).

### Statistical Analysis

Descriptive statistics of characteristics are described as mean ± SD or % for frequencies; in skewed distributions, a median (range) was provided at year 7 and 15. Unadjusted Pearson correlation coefficients were calculated between CAMs and cognitive function test scores. Unadjusted correlation coefficients between repeated measures of CAMs were calculated. Multiple linear regression modeled the associations for each of the five CAMs at years 7 and 15 with cognitive function measured by RAVLT, DSST, and Stroop Test in year 25. Test for trend used multivariable linear regression models with continuous CAM concentration as predictors was also provided. To more fully describe the findings and to study the appropriateness of treating the CAM as a continuous variable, each of the five CAMs was categorized into quartiles. A 3 degree of freedom *p* value based on an *F* test for any difference in cognitive test scores among the quartiles was computed. Model 1 was adjusted for age, sex, race (white, black), educational attainment, and study center (Birmingham, Chicago, Minneapolis, and Oakland). We further adjusted for lifestyle behaviors including smoking, alcohol intake, physical activity, and *a priori* diet quality score in model 2 and were further adjusted for physical factors, BMI, elevated blood pressure (including use of antihypertensive medication), diabetes, blood lipids, and C-reactive protein in model 3. Because the physical factors may be in the pathway linking CAMs to cognitive test scores, we regarded model 2 as deconfounding and model 3 as explanatory of mechanisms by which CAMs may relate to cognitive function test scores.

Statistical testing was two-sided with type 1 error rate 0.05. Statistical analyses were conducted using SAS 9.3 (SAS Institute Inc., Cary, NC, USA).

## Results

### Sample Characteristics and Change with Aging

Among the 2,690 participants included in year 7 analysis, the average age was 32 years; 42% were black and 45% were males; 23% were current smokers, less than 1% were diabetic, and 16% had elevated blood pressure (including use of antihypertensive medication) (Table 1).

**Table 1:** **Participant characteristics with circulating biomarkers measurements of endothelial function in 1992–1993 and in 2000–2001, and with cognitive measurement in 2010–2011: Coronary Artery Risk Development in Young Adults**.

	Year 7 (1992–1993)	Year 15 (2000–2001)
Participants (*n*)	2,690	2,848
Age (years)	32.2 ± 3.6	40.3 ± 3.6
Race (% Black)	42.2	43.5
Sex (% male)	44.7	43.3
Education (years)	14.9 ± 2.5	15.1 ± 2.5
Alcohol (mL/day)^a^	2.43 (0, 13.00)	2.43 (0, 13.29)
**Smoking**		
Current (%)	23.0	19.8
Former (%)	17.3	18.4
Physical activity (exercise units)^a^	270 (140, 487.0)	288 (144, 498)
Body mass index (kg/m^2^)	26.5 ± 5.9	28.6 ± 6.8
Diabetes (%)	0.82	3.72
**Blood pressure**		
Elevated blood pressure (≥130/85 mmHg) (%)	15.5	29.5
**Blood lipids**		
HDL cholesterol (mg/dL)	51.9 ± 13.4	50.9 ± 14.3
LDL cholesterol (mg/dL)	108.0 ± 31.0	112.8 ± 31.5
Triglycerides (mg/dL)	81.1 ± 52.2	98.5 ± 59.8
Dietary score^b^ (score)	67.5 ± 12.1	not done
**Endothelial function biomarkers (soluble form)**		
ICAM-1 (ng/mL)	138.1 ± 30.8	152.7 ± 41.4
VCAM-1 (ng/mL)	528.1 ± 155.3	528.0 ± 166.4
P-selectin (ng/mL)	28.2 ± 9.3	36.7 ± 11.2
E-selectin (ng/mL)	33.1 ± 14.6	34.4 ± 13.4
Fractalkine (ng/mL)	0.54 ± 0.30	0.58 ± 0.17
**Inflammatory biomarker**		
C-reactive protein (ng/ml)	2.7 ± 7.9	2.0 ± 2.7
**Cognitive function test**		
Rey Auditory Verbal Learning Test (words)	8.5 ± 3.2	8.5 ± 3.2
Digit Symbol Substitution Test (symbols)	70.9 ± 15.8	70.8 ± 15.8
Stroop Test (seconds + errors)	44.4 ± 12.9	44.6 ± 12.9

*Data are presented as mean ± SD*.

*^a^Data displayed are median (interquartile range)*.

*^b^Dietary score was not measured in year 15*.

*ICAM-1, intercellular adhesion molecule-1; VCAM-1, vascular cellular adhesion molecule-1*.

The years of educational attainment, amount of physical activity, BMI, and concentrations of LDL cholesterol, triglycerides, and ICAM-1, VCAM-1, P-selectin, E-selectin, and fractalkine all increased in year 15 compared to year 7. The average year 7 *a priori* diet quality score was 68. Correlation between year 7 and 15 repeated measures was 0.66 for ICAM-1, 0.79 for VCAM-1, 0.65 for P-selectin, 0.82 for E-selectin, and 0.45 for fractalkine. Correlations of CAMs measured in year 7 or year 15 with each other were 0.26–0.43 for pairs of ICAM-1, P-selectin, and E-selectin, and lower for other combinations. Some aspects of the association of the CAMs have been reported previously (22, 23). In year 7, less educational attainment, less physical activity, and lower quality diet were associated with higher ICAM-1, P-selectin, and E-selectin concentrations, while current smoking, higher BMI, hypertension, adverse blood lipid profile, and higher C-reactive protein were associated with higher concentrations of these CAMs. Associations of covariables with VCAM-1 concentrations were often opposite in direction to the associations with ICAM-1, P-selectin, and E-selectin concentrations. Fractalkine concentration had low positive correlations with alcohol intake and physical activity, but other covariables had little correlation with fractalkine concentrations (data not shown). Race and sex differences (indicated for year 7 as correlations in Table 2) have not previously been reported. ICAM-1, E-selectin, and fractalkine had higher concentrations in blacks (149.8 ± 25.8, 35.6 ± 14.8, and 0.55 ± 0.40, respectively, in year 7, 163.5 ± 43.1, 37.1 ± 13.0, and 0.59 ± 0.18, respectively, in year 15) compared to whites (132.2 ± 32.7, 31.6 ± 14.2, and 0.53 ± 0.15 in year 7, and 145.2 ± 36.1, 32.0 ± 12.5, and 0.57 ± 0.14 in year 15). P-selectin was higher in blacks in year 7 only (28.7 ± 9.1) compared to whites (28.0 ± 9.3). VCAM-1 was higher among whites in years 7 and 15 (570.0 ± 149.8 and 568.8 ± 156.0, respectively) compared to blacks (471.3 ± 143.7 and 477.0 ± 155.9, respectively). Men had a higher concentration of P-selectin (year 7: 31.1 ± 0.3, year 15: 39.4 ± 10.8), E-selectin (year 7: 36.8 ± 14.3, year 15: 37.3 ± 13.1), and fractalkine (year 7: 0.57 ± 0.40, year 15: 0.59 ± 0.10) in either year compared to women (year 7: 26.1 ± 8.2, year 15: 34.4 ± 10.2 for P-selectin, year 7: 30.3 ± 14.0, year 15: 31.8 ± 12.4 for E-selectin, and year 7: 0.52 ± 0.10, year 15: 0.57 ± 0.17 for fractalkine). ICAM-1 and VCAM-1 did not differ between sexes in either year.

**Table 2:** **Pearson correlation coefficients between year 7 circulating cellular adhesion molecules and other variables measured at year 7**.

	ICAM-1	VCAM-1	P-selectin	E-selectin	Fractalkine
Age (years)	−0.03	0.06*	0.05*	0.05	−0.02
White race	−0.28*	0.32*	−0.04	−0.14*	−0.04
Female	−0.03	−0.04	−0.28*	−0.22*	−0.09*
Education (years)	−0.26*	0.16*	−0.10*	−0.17*	−0.02
Alcohol (mL/day)	−0.01	−0.06*	0.09*	0.11*	0.07*
Current smoking	0.31*	−0.10*	0.07*	0.07*	−0.04
Physical activity (exercise units)	0.14*	0.03	0.05*	<0.01	0.07*
Body mass index (kg/m^2^)	0.24*	−0.15*	0.13*	0.28*	−0.04
Diabetes	<0.01	<0.01	0.07*	0.06*	0.01
Blood pressure (mmHg)	0.10*	−0.06*	0.14*	0.16*	0.06*
**Blood lipids**					
HDL cholesterol (mg/dL)	−0.19*	−0.03	−0.15*	−0.20*	<0.01
LDL cholesterol (mg/dL)	0.13*	−0.13*	0.14*	0.13*	0.03
Triglycerides (mg/dL)	0.15*	−0.07*	0.20*	0.24*	<0.01
Dietary score (score)	−0.26*	0.11*	−0.14*	−0.19*	−0.04

*ICAM-1, intercellular adhesion molecule-1; VCAM-1, vascular cellular adhesion molecule-1*.

*Significant correlation (*p* ≤ 0.01).

### CAMs and Cognitive Function: Adjusted Models

Participants in the fourth (highest) quartile of year 7 ICAM-1 concentration had worse cognitive function tests in RAVLT, DSST, and Stroop Tests compared to those in the first (lowest) quartile of ICAM-1 concentration after adjusting for demographic and lifestyle behaviors (β = −0.56, β = −3.70, and β = 2.67, all *p* value < 0.05, respectively, Table 3, model 2). The associations with DSST and Stroop Tests looked like threshold relationships. With further adjustment for physical variables (Table 3, model 3), the association among higher ICAM-1 and RAVLT and DSST cognitive test scores was attenuated yet remained significant (fourth vs. first quartile of ICAM-1: RAVLT β = −0.47, *p* = 0.04, DSST β = −2.28, *p* = 0.03), while the association between CAMs and Stroop Test were attenuated to non-significance. None of the cognitive function test scores were significantly associated with VCAM-1, P-selectin, E-selectin, or fractalkine measured in year 7 in model 2 (Table S1 in Supplementary Material).

**Table 3:** **Regression coefficients (95% CI) of year 25 cognitive test per year 7 intercellular adhesion molecule-1 (ICAM-1) (*N* = 1,798 for ICAM-1)**.

	Quartiles of ICAM-1 (ng/ml)				*p* Value (3 df)	*p* Trend
	Q1 (*N* = 449) 62.69–117.81	Q2 (*N* = 450) 117.82–135.35	Q3 (*N* = 450) 135.36–152.80	Q4 (*N* = 459) 152.81–362.87		
**Rey Auditory Verbal Learning Test**						
Model 1^a^	0	−0.19 (−0.56, 0.18)	−0.42 (−0.81, −0.03)	−0.61 (−1.02, −0.20)	0.02	0.01
Model 2^b^	0	−0.19 (−0.56, 0.18)	−0.41 (−0.82, 0.00)	−0.56 (−0.99, −0.13)	0.05	0.03
Model 3^c^	0	−0.15 (−0.52, 0.22)	−0.39 (−0.80, 0.02)	−0.47 (−0.92, −0.02)	0.14	0.09
**Digit Symbol Substitution Test**						
Model 1^a^	0	−1.09 (−2.85, 067)	−0.03 (−1.87, 1.81)	−3.70 (−2.25, 1.51)	<0.01	<0.01
Model 2^b^	0	−0.88 (−2.64, 0.88)	0.36 (−1.50, 2.22)	−2.71 (−4.69, −0.73)	<0.01	<0.01
Model 3^c^	0	−0.77 (−2.53, 0.99)	0.52 (−1.38, 2.42)	−2.28 (−4.34, −0.22)	0.02	0.01
**Stroop Test**						
Model 1^a^	0	0.03 (−1.48, 1.54)	0.32 (−1.27, 1.91)	2.67 (1.06, 4.28)	<0.01	<0.01
Model 2^b^	0	−0.14 (−1.65, 1.37)	0.03 (−1.56, 1.62)	1.87 (0.18, 3.56)	0.05	<0.01
Model 3^c^	0	−0.29 (−1.80, 1.22)	−0.24 (−1.87, 1.39)	1.29 (−0.47, 3.05)	0.19	0.01

*Each regression coefficient is the mean cognitive test score difference from the cognitive test score in quartile 1 of the given cellular adhesion molecule (CAM). *p* Trend is computed across the continuous CAM variable*.

*^a^Model 1: adjusted for age, race, sex, education, and center*.

*^b^Model 2: model 1 + smoking, alcohol, physical activity, and a priori diet quality score*.

*^c^Model 3: model 2 + body mass index, elevated blood pressure (including use of antihypertensive medication), diabetes, blood lipids, and C-reactive protein*.

Findings were of similar strength and significance for year 15 CAMs predicting year 25 cognitive function test scores. After adjusting for demographic and lifestyle behaviors, higher concentration of ICAM-1 was associated with worse DSST (β = −2.51 *p* < 0.01) and Stroop Test scores (β = 1.65, *p* = 0.01) (Table 4, model 2). The association with Stroop Test was attenuated to non-significance after physical variables were adjusted.

**Table 4:** **Regression coefficients (95% CI) of year 25 cognitive test per year 15 intercellular adhesion molecule-1 (ICAM-1) and E-selectin (*N* = 2,665 for ICAM-1)**.

	Quartiles of ICAM-1 (ng/ml)				*p* Value (3 df)	*p* Trend
	Q1 (*N* = 666) 55.85–125.51	Q2 (*N* = 666) 125.52–146.11	Q3 (*N* = 667) 146.12–169.75	Q4 (*N* = 666) 169.76–534.56		
**Rey Auditory Verbal Learning Test**						
Model 1^a^	0	−0.08 (−0.39, 0.23)	−0.01 (−0.32, 0.30)	−0.14 (−0.47, 0.19)	0.81	0.33
Model 2^b^	0	−0.09 (−0.40, 0.22)	0.00 (−0.31, 0.31)	−0.09 (−0.42, 0.24)	0.88	0.56
Model 3^c^	0	−0.09 (−0.40, 0.22)	0.00 (−0.31, 0.31)	−0.05 (−0.40, 0.30)	0.93	0.91
**Digit Symbol Substitution Test**						
Model 1^a^	0	−1.83 (−3.26, −0.40)	−2.23 (−3.68, −0.78)	−3.41 (−4.92, −1.90)	<0.01	<0.01
Model 2^b^	0	−1.79 (−3.22, −0.36)	−1.98 (−3.43, −0.53)	−2.51 (−4.06, −0.96)	0.01	<0.01
Model 3^c^	0	−1.65 (−3.08, −0.22)	−1.61 (−3.09, −0.12)	−1.72 (−3.37, −0.07)	0.08	<0.01
**Stroop Test**						
Model 1^a^	0	1.33 (0.11, 2.54)	1.52 (0.29, 2.75)	2.01 (0.74, 3.28)	0.01	<0.01
Model 2^b^	0	1.33 (0.11, 2.54)	1.43 (0.20, 2.66)	1.65 (0.34, 2.96)	0.05	<0.01
Model 3^c^	0	1.19 (−0.04, 2.42)	1.10 (−0.17, 2.37)	0.89 (−0.50, 2.28)	0.23	0.05

*Each regression coefficient is the mean cognitive test score difference from the cognitive test score in quartile 1 of the given cellular adhesion molecule (CAM). *p* Trend is computed across the continuous CAM variable*.

*^a^Model 1: adjusted for age, race, sex, education, and center*.

*^b^Model 2: model 1 + smoking, alcohol, physical activity, and a priori diet quality score*.

*^c^Model 3: model 2 + body mass index, elevated blood pressure (including use of antihypertensive medication), diabetes, blood lipids, and C-reactive protein*.

In a sensitivity analysis, the regression coefficients were similar in magnitude when the analysis was restricted to 1,017 participants who had all CAMs measured at both year 7 and year 15 or restricting to those who had both measures of a particular CAM (ICAM-1 *n* = 1,516; VCAM-1 *n* = 2,245; P-selectin *n* = 2,247; E-selectin *n* = 1,721; fractalkine *n* = 2,039). Several other sensitivity analyses were done (data not shown). The change in ICAM-1 measured in year 15 minus year 7 was available in 1,516 participants but was not significantly associated with any of the year 25 cognitive test scores. However, year 7 ICAM-1 remained significantly associated as in Table 2 when added to a model which included ICAM-1 change. Cognitive function was not related to change in the other CAMs or fractalkine. Findings were similar to those reported here using the average of year 7 and year 15 CAMs. The sum of standardized deviates of all the CAMs and fractalkine was not related to cognitive function.

## Discussion

This study provides support for the concept that endothelial dysfunction (measured at age 25–37 years old) is associated with cognitive function measured years later in middle age (43–55 years old). People in the highest vs. lowest quartile of ICAM-1 in years 7 and 15 (average ages 32 and 40) had worse cognitive test scores related to psychomotor speed and attention (DSST) and executive function (Stroop Test) measured at average age 50, and similarly for ICAM-1 measured at year 15 (average age 40). RAVLT (short-term memory) was associated with worse ICAM-1 in year 7. Other circulating CAMs (years 7 and 15), namely, VCAM-1, P-selectin, E-selectin, and the chemokine fractalkine were not associated with cognitive function. The association with ICAM-1 might be of a threshold type, given that the highest quartile of year 7 ICAM-1 was significantly associated with worse cognitive test scores in each test compared to the lowest ICAM-1 quartile, with little gradient in-between; however, findings with year 15 ICAM-1 were more graded. Adjustment for physical factors including BMI, elevated blood pressure, diabetes, blood lipids, and C-reactive protein attenuated the association between ICAM-1 and cognitive test scores, mostly to non-significance, evidenced based on the 3 degree of freedom test; we consider this attenuation to be explanatory, in that these physical factors could be on a pathway between ICAM-1 and cognitive test scores.

Although vascular, neurovascular, and endothelial risk factors measured at the cellular level are associated with dementia in elderly populations (24–38), inconsistent associations have been reported between plasma endothelial dysfunction markers and the risk of cognitive impairment (39–41). Circulating CAMs are easy to measure in human subjects and are commonly used as markers of endothelial dysfunction. Circulating ICAM-1, P-selectin, and E-selectin are correlated with each other, as expected (4–8). In the present paper, ICAM-1 was the only circulating CAM that was consistently associated with worse cognitive test scores. In earlier studies, ICAM-1 predicted presence of coronary artery calcification (other CAMs were not studied) (23), and only ICAM-1 and E-selectin predicted future diabetes (22). Circulating CAMs appear not to capture some known characteristics of CAMs as they interact with each other in the endothelium; therefore, the levels of circulating CAMs may be less sensitive indicators than cellular CAMs. The observations reported here should be interpreted with caution and may not eliminate the possibility that associations of cognitive function exist with endothelial function in the endothelium itself.

A number of studies suggest that cerebrovascular dysfunction plays a role in the development of neurodegenerative diseases (11), including decline in cognitive function. Epidemiological studies including the Honolulu–Asia Study (24), Rotterdam Study (25, 26), EURODEM (27), and Kungsholmen Project (28) report several vascular risk factors such as high blood pressure, diabetes mellitus, atherosclerosis, apolipoprotein E, heart failure, and recent strokes being associated with dementia, which causes cerebral hypoperfusion (24, 26, 28). Similarly, the Framingham study reports general cardiovascular disease and stroke risk scores to be correlated with cognitive decline in late middle age. Thus, studies suggest cardiovascular disease and stroke risk scores to be targetable risk factors for assessing risk of cognitive dysfunction (29). Other current literature suggests endothelial dysfunction is involved in the pathogenesis of neurodegenerative diseases and Alzheimer’s disease (11). Therefore, if endothelial dysfunction, measured by circulating CAMs, is associated with worse cognitive test scores, endothelial function may represent a possible modifiable risk factor to prevent Alzheimer’s disease and/or to delay cognitive decline.

A prospective study with 452 Scottish men and women initially aged 73 years who were followed for 16 years and had measurements of ICAM-1, VCAM-1, and E-selectin showed similar results indicating ICAM-1 was associated with worse cognitive function test scores especially with the speed of processing information and executive function of frontal lobe. Similar to our study, the Edinburgh Artery Study reported no association of VCAM-1 and E-selectin with verbal memory, the speed of processing information, and executive function (42). A population-based cohort study with 377 participants which combined vWF, ICAM-1, VCAM-1, thrombomodulin, and E-selectin to create an overall index of endothelial dysfunction reported an inverse association between these endothelial dysfunction markers and information processing speed/attention and executive functioning (43), corresponding to DSST and Stroop Test in our study. This study did not report findings for individual CAMs. No other studies have looked at the associations of P-selectin and fractalkine with cognitive function.

A strength of our study is use of a large community-based prospective cohort including more than 2,500 healthy middle-aged participants, allowing generalization of the study results to middle-aged US whites and African Americans. Additionally, the age of our subjects when cognitive function tests were performed ranged from 43 to 55 years old, younger than in most other studies where the age was around 70s at baseline.

A limitation of our study is that we did not have the same participants in years 7 and 15; however, this could be seen as a strength, because our findings were robust regardless of whether the CAMs were obtained at either CARDIA year 7 or 15, or restricting it to people who had both measures. Furthermore, CARDIA does not have baseline data for the cognitive function tests used at year 25, which makes it difficult to determine the temporality and inference about the causality between the markers of endothelial dysfunction and cognitive function. Therefore, further studies with longitudinal cognitive function data are needed to explore the temporal association between endothelial dysfunction and cognitive performance among middle-aged healthy participants. The restriction of our sample size to 1,516 for study of 8 year change in ICAM-1 may have contributed to lack of association of CAM change with any of the year 25 cognitive test scores. Furthermore, as is the case in all observational epidemiologic studies, our study is limited by potential residual confounding from unmeasured or unknown confounders, which could result in false positive findings.

## Conclusion

In conclusion, our study provides results consistent with other studies finding that higher concentration of ICAM-1 (particularly in its highest quartile) is associated with worse cognitive function test scores. These lower cognitive test scores in middle age are of interest because they may be on a trajectory to cognitive impairment, although the idea of a trajectory must be confirmed in future study. The concept that vascular health is associated with subclinically lower cognitive scores, as well as with frank dementia, is of substantial interest. With the increasing prevalence of older adults with cognitive deficits, along with the Healthy People 2020s goal, it is important to improve the identification of and care for those who are at risk.

## Ethics Statement

This study was carried out in accordance with the recommendations of CARDIA’s Institutional Review Boards. All subjects gave written, signed, informed consent at each clinic examination in accordance with the Declaration of Helsinki. The protocol was approved by the CARDIA Institutional Review Board.

## Author Contributions

CY: writing and data analysis; LS, AO, and SS: writing and critical review of the manuscript; MG, LL, and OS: critical review of the manuscript; AR: funding and critical review of the manuscript; KY: conception, interpretation, critical review of the manuscript, and funding; DJ: supervision, concept, data analysis, writing, critical review of the manuscript, and funding.

## Conflict of Interest Statement

CY, LS, MG, LL, AO, AR, OS, SS, and DJ report no disclosures. KY has served on data safety monitoring boards for Takeda Inc., Pfizer Inc., and Medivation Inc. and served as a consultant for Novartis Inc.

## References

[1] Smith RG Betancourt L Sun Y. Molecular endocrinology and physiology of the aging central nervous system. Endocr Rev (2005) 26:203–50. 10.1210/er.2002-0017 15561803

[2] Fotuhi M Hachinski V Whitehouse PJ. Changing perspectives regarding late-life dementia. Nat Rev Neurol (2009) 5:649–58. 10.1038/nrneurol.2009.175 19918254

[3] Plassman BL Langa KM Fisher GG Heeringa SG Weir DR OFstedal MB Prevalence of dementia in the United States: the aging, demographics, and memory study. Neuroepidemiology (2007) 29:125–32. 10.1159/000109998 17975326 PMC2705925

[4] Price DT Loscalzo J Cellular adhesion molecules and atherogenesis. Am J Med (2003) 107(1):85–97. 10.1016/S0002-9343(99)00153-9 10403357

[5] Hwang SJ Ballantyne CM Sharrett AR Smith LC Davis CE Gotto AM Jr Circulating adhesion molecules VCAM-1, ICAM-1, and E-selectin in carotid atherosclerosis and incident coronary heart disease cases: the Atherosclerosis Risk In Communities (ARIC) study. Circulation (1997) 16(96):4219–25. 10.1161/01.CIR.96.12.4219 9416885

[6] Goda S Imai T Yoshie O Yoneda O Inoue H Nagano Y CX3C-chemokine, fractalkine-enhanced adhesion of THP-1 cells to endothelial cells through integrin-dependent and -independent mechanisms. J Immunol (2000) 15(164):4313–20. 10.4049/jimmunol.164.8.4313 10754331

[7] Deanfield JE Halcox JP Rabelink TJ Endothelial function and dysfunction: testing and clinical relevance. Circulation (2007) 115(10):1285–95. 10.1161/CIRCULATIONAHA.106.652859 17353456

[8] Khazaei M Moien-Afshari F Laher I Vascular endothelial function in health and diseases. Pathophysiology (2008) 15:49–67. 10.1016/j.pathophys.2008.02.002 18434105

[9] Versari D Daghini E Virdis A Ghiadoni L Taddei S Endothelial dysfunction as a target for prevention of cardiovascular disease. Diabetes Care (2009) 32(Suppl 2):S314–21. 10.2337/dc09-S330 19875572 PMC2811443

[10] Zlokovic BV. Neurovascular pathways to neurodegeneration in Alzheimer’s disease and other disorders. Nat Rev Neurosci (2011) 3(12):723–38. 10.1038/nrn3114 22048062 PMC4036520

[11] Kelleher RJ Soiza RL. Evidence of endothelial dysfunction in the development of Alzheimer’s disease: is Alzheimer’s a vascular disorder? Am J Cardiovasc Dis (2013) 3(4):197–226. 24224133 PMC3819581

[12] Gonzales MM Tarumi T Tanaka H Sugawara J Swann-Sternberg T Goudarzi K Functional imaging of working memory and peripheral endothelial function in middle-aged adults. Brain Cogn (2010) 73:146–51. 10.1016/j.bandc.2010.04.007 20493622 PMC2891094

[13] Friedman GD Cutter GR Donahue RP Hughes GH Hulley SB Jacobs DR Jr CARDIA: study design, recruitment, and some characteristics of the examined subjects. J Clin Epidemiol (1988) 41:1105–16. 10.1016/0895-4356(88)90080-7 3204420

[14] Sijtsma FP Meyer KA Steffen LM Van Horn L Shikany JM Odegaard AO Diet quality and markers of endothelial function: the CARDIA study. Nutr Metab Cardiovasc Dis (2014) 24:632–8. 10.1016/j.numecd.2013.12.010 24534074 PMC4037360

[15] Schmidt M Rey Auditory Verbal Learning Test: A Handbook. Los Angeles: Western Psychological Services (1996).

[16] Wechsler D Wechsler Adult Intelligence Scale – III (WAIS-III) Administration and Scoring Manual. 3rd ed San Antonio, TX: The Psychological Corporation (1997).

[17] Stroop J Studies of interference in serial verbal reaction. J Exp Psychol (1935) 18:643–62. 10.1037/h0054651

[18] Sijtsma FP Meyer KA Steffen LM Shikany JM Van Horn L Harnack L Longitudinal trends in diet and effects of sex, race, and education on dietary quality score change: the Coronary Artery Risk Development in Young Adults study. Am J Clin Nutr (2012) 95:580–6. 10.3945/ajcn.111.020719 22301926 PMC3278239

[19] Lakoski SG Herrington DM Siscovick DM Hulley SB. C-reactive protein concentration and incident hypertension in young adults: the CARDIA study. Arch Intern Med (2006) 166:345–9. 10.1001/archinte.166.3.345 16476876

[20] Carnethon MR Sternfeld B Schreiner PJ Jacobs DR Jr Lewis CE Liu K Association of 20 year changes in cardiorespiratory fitness with incident type 2 diabetes: the coronary artery risk development in young adults (CARDIA) fitness study. Diabetes Care (2009) 32:1284–8. 10.2337/dc08-1971 19324945 PMC2699748

[21] Zhu N Jacobs DR Meyer KA He K Launer L Reis JP Cognitive function in a middle aged cohort is related to higher quality dietary pattern 5 and 25 years earlier: the CARDIA study. J Nutr Health Aging (2015) 19:33–8. 10.1007/s12603-014-0491-7 25560814 PMC5466430

[22] Odegaard AO Jacobs DR Jr Sanchez OA Goff DC Jr Reiner AP Gross MD. Oxidative stress, inflammation, endothelial dysfunction and incidence of type 2 diabetes. Cardiovasc Diabetol (2016) 15:51. 10.1186/s12933-016-0369-6 27013319 PMC4806507

[23] Gross MD Bielinski SJ Suarez-Lopez JR Reiner AP Bailey K Thyagarajan B Circulating soluble intercellular adhesion molecule 1 and subclinical atherosclerosis: the Coronary Artery Risk Development in Young Adults study. Clin Chem (2012) 58(2):411–20. 10.1373/clinchem.2011.168559 22179741 PMC3867124

[24] Launer LJ Ross GW Petrovitch H Masaki K Foley D White LR Midlife blood pressure and dementia: the Honolulu-Asia aging study. Neurobiol Aging (2000) 21:49–55. 10.1016/S0197-4580(00)00096-8 10794848

[25] Ott A Stolk RP Hofman A van Harskamp F Grobbee DE Breteler MM. Association of diabetes mellitus and dementia: the Rotterdam study. Diabetologia (1996) 39:1392–7. 10.1007/s001250050588 8933010

[26] Hofman A Ott A Breteler MM Bots ML Slooter AJ van Harskamp F Atherosclerosis, apolipoprotein E, and prevalence of dementia and Alzheimer’s disease in the Rotterdam study. Lancet (1997) 18(349):151–4. 10.1016/S0140-6736(96)09328-2 9111537

[27] Launer LJ Andersen K Dewey ME Letenneur L Ott A Amaducci LA Rates and risk factors for dementia and Alzheimer’s disease: results from EURODEM pooled analyses. EURODEM Incidence Research Group and Work Groups. European Studies of Dementia. Neurology (1999) 1(52):78–84. 10.1212/WNL.52.1.78 9921852

[28] Fratiglioni L Winblad B von Strauss E Prevention of Alzheimer’s disease and dementia. Major findings from the Kungsholmen project. Physiol Behav (2007) 10(92):98–104. 10.1016/j.physbeh.2007.05.059 17588621

[29] Kaffashian S Dugravot A Elbaz A Shipley MJ Sabia S Kivimaki M Predicting cognitive decline: a dementia risk score vs. the Framingham vascular risk scores. Neurology (2013) 2(80):1300–6. 10.1212/WNL.0b013e31828ab370 23547265 PMC3656460

[30] Breteler MM Vascular risk factors for Alzheimer’s disease: an epidemiologic perspective. Neurobiol Aging (2000) 21:153–60. 10.1016/S0197-4580(99)00110-4 10867200

[31] de la Torre JC Mussivand T. Can disturbed brain microcirculation cause Alzheimer’s disease? Neurol Res (1993) 15:146–53. 10.1080/01616412.1993.11740127 8103579

[32] Iadecola C Gorelick PB Converging pathogenic mechanisms in vascular and neurodegenerative dementia. Stroke (2003) 34:335–7. 10.1161/01.STR.0000054050.51530.76 12574528

[33] Kalaria RN. The role of cerebral ischemia in Alzheimer’s disease. Neurobiol Aging (2000) 21:321–30. 10.1016/S0197-4580(00)00125-1 10867217

[34] Zuliani G Cavalieri M Galvani M Passaro A Munari MR Bosi C Markers of endothelial dysfunction in older subjects with late onset Alzheimer’s disease or vascular dementia. J Neurol Sci (2008) 272:164–70. 10.1016/j.jns.2008.05.020 18597785

[35] Smith C Carney J Starke-Reed P Oliver CN Stadtman ER Floyd RA Excess brain protein oxidation and enzyme dysfunction in normal aging and in Alzheimer disease. Proc Natl Acad Sci U S A (1991) 88:10540–3. 10.1073/pnas.88.23.10540 1683703 PMC52964

[36] Stadtman ER. Protein oxidation in aging and age-related diseases. Ann N Y Acad Sci (2001) 928:22–38. 10.1111/j.1749-6632.2001.tb05632.x 11795513

[37] Schmidt R Schmidt H Curb J Masaki K White LR Launer LJ. Early inflammation and dementia: a 25 year follow-up of the Honolulu-Asia Aging Study. Ann Neurol (2002) 52:168–74. 10.1002/ana.10265 12210786

[38] Ravaglia G Forti P Maioli F Chiappelli M Montesi F Tumini E Blood inflammatory markers and risk of dementia: the Conselice study of brain aging. Neurobiol Aging (2007) 28:1810–20. 10.1016/j.neurobiolaging.2006.08.012 17011077

[39] Hochstrasser T Weiss E Marksteiner J Humpel C. Soluble cell adhesion molecules in monocytes of Alzheimer’s disease and mild cognitive impairment. Exp Gerontol (2010) 45:70–4. 10.1016/j.exger.2009.10.005 19836440 PMC4311053

[40] Tan Z Beiser A Vasan R Roubenoff R Dinarello CA Harris TB Inflammatory markers and the risk of Alzheimer disease: the Framingham study. Neurology (2007) 68:1902–8. 10.1212/01.wnl.0000263217.36439.da 17536046

[41] Obasi CN Cruickshanks KJ Nondahl DM Klein BE Klein R Nieto FJ Association of biomarkers for inflammation, endothelial dysfunction and oxidative stress with cognitive impairment. The epidemiology of hearing loss study (EHLS). Oxid Antioxid Med Sci (2012) 1(3):169–73. 10.5455/oams.031212.br.004 23814681 PMC3693393

[42] Rafnsson SB Deary IJ Smith FB Whiteman MC Rumley A Lowe GD Cognitive decline and markers of inflammation and hemostasis: the Edinburgh Artery study. J Am Geriatr Soc (2007) 55:700–7. 10.1111/j.1532-5415.2007.01158.x 17493189

[43] Heringa SM van den Berg E Reijmer YD Nijpels G Stehouwer CDA Schalkwijk CG Markers of low-grade inflammation and endothelial dysfunction are related to reduced information processing speed and executive functioning in an older population – the Hoorn study. Psychoneuroendocrinology (2014) 40:108–11. 10.1016/j.psyneuen.2013.11.011 24485482

