# Supplementary material for: Circulating Cellular Adhesion Molecules and Cognitive Function: The Coronary Artery Risk Development in Young Adults Study
Source: Front Cardiovasc Med. 2017 May 24;4:37. doi: 10.3389/fcvm.2017.00037 (PMC5442165; doi:10.3389/fcvm.2017.00037)
Supplement: Supplementary file 1 [file data_sheet_1.doc]

Supplementary Material

**CELLULAR ADHESION MOLECULES AND COGNITION**

**Cynthia Yoon^1^, Lyn M Steffen^1^, Myron D. Gross ^1^, Lenore J Launer ^2^, Andrew Odegaard ^3^, Alexander Reiner^4^, Otto Sanchez ^1^, Kristine Yaffe ^5^, Stephen Sidney ^6^, David R Jacobs Jr ^1^***

**Correspondence:** David R. Jacobs Jr, [jacob004@umn.edu](mailto:jacob004@umn.edu)

# Supplementary Figure

1029 did not attend year 7

1 drop out

54 stroke or TIA between years 0 and 25

1172 no CAM measured at year 7

1759 no cognitive test measured at year 25

1523 missing covariates at year 7

5115 participants

2690 participants included in year 7

1443 did not attend year 15

1 drop out

54 stroke or TIA between years 0 and 25

816 no CAM measured at year 15

1759 no cognitive test measured at year 25

1588 missing covariates at year 15

5115 participants

2848 participants included in year 15

**Supplementary Figure 1.** Flow Chart for the study with cellular adhesion molecules measured at Years 7 or 15 and cognitive performance assessed at Year 25, with sample size for the analyses

**Online Table 1. Regression coefficients (95%CI) of year 25 cognitive test per year 7 VCAM-1, P selectin, and Fractalkine. (N=2,655 for VCAM-1, N=2,662 for P-selectin, N=2,083 for E-selectin, N=2,468 for fractalkine)**

|  | Quartiles of VCAM-1 (ng/ml) | | | | | P value (3 df) | P trend |
| --- | --- | --- | --- | --- | --- | --- | --- |
|  | Q1 (N=663) | Q2 (N=664) | Q3 (N=664) | Q4 (N=664) | |  |  |
|  | 177.89—416.71 | 416.72—506.85 | 506.86—615.87 | 615.88—1357.16 | |  |  |
|  | Rey Auditory Verbal Learning Test (RAVLT) | | | | | | |
| Model 1* | 0 | 0.18  (−0.13,0.49) | 0.23  (−0.08,0.54) | 0.15  (−0.18,0.48) | | 0.52 | 0.22 |
| Model 2┼ | 0 | 0.15  (−0.16, 0.46) | 0.20  (−0.11, 0.51) | 0.11  (−0.22, 0.44) | | 0.65 | 0.32 |
| Model 3‡ | 0 | 0.13  (−0.18,0.44) | 0.16  (−0.15,0.47) | 0.05  (−0.28,0.38) | | 0.74 | 0.50 |
|  | Digit Symbol Substitution Test (DSST) | | | | | | |
| Model 1* | 0 | 1.41  (−0.06,2.88) | 1.09  (−0.40,2.57) | 0.55  (−0.98, 2.08) | | 0.25 | 0.84 |
| Model 2┼ | 0 | 1.22  (−0.23, 2.67) | 0.87  (−0.58, 2.32) | 0.26  (−1.27, 1.79) | | 0.33 | 0.53 |
| Model 3‡ | 0 | 1.15  (−0.30, 2.60) | 0.81  (−0.70, 2.32) | 0.17  (−1.38, 1.72) | | 0.36 | 0.48 |
|  | Stroop Test | | | | | | |
| Model 1* | 0 | −1.14  (−2.39,0.11) | −0.85  (−2.12,0. 42) | −1.19  (−2.50, 0.12) | 0.24 | | 0.20 |
| Model 2┼ | 0 | −1.02  (−2.29, 0.25) | −0.69  (−1.96, 0.58) | −1.06  (−2.37, 0.25) | 0.35 | | 0.28 |
| Model 3‡ | 0 | −0.92  (−2.17, 0.33) | −0.49  (−0.78, 0.80) | −0.78  (−2.11, 0.55) | 0.51 | | 0.48 |
|  |  | | | |  | |  |
|  | Quartiles of P-selectin (ng/ml) | | | | P value (3df) | | P trend |
|  | Q1 (N=665) | Q2 (N=665) | Q3 (N=666) | Q4 (N=666) |  | |  |
|  | 5.66-21.91 | 21.92-27.39 | 27.40-33.42 | 33.43-124.63 |  | |  |
|  | Rey Auditory Verbal Learning Test (RAVLT) | | | | | | |
| Model 1* | 0 | 0.06  (−0.25,0.37) | −0.24  (−0.55,0.07) | −0.10  (−0.41,0.21) | | 0.25 | 0.55 |
| Model 2┼ | 0 | 0.09  (−0.22, 0.40) | −0.19  (−0.50, 0.12) | −0.05  (−0.36, 0.26) | | 0.37 | 0.74 |
| Model 3‡ | 0 | 0.11  (−0.20,0.42) | −0.18  (−0.49,0.13) | 0.04  (−0.29,0.37) | | 0.29 | 0.93 |
|  | Digit Symbol Substitution Test (DSST) | | | | | | |
| Model 1* | 0 | 0.52  (−0.93,1.97) | −0.14  (−1.61,1.33) | −0.26  (−1.79, 1.27) | | 0.75 | 0.40 |
| Model 2┼ | 0 | 0.76  (−0.69. 2.21) | 0.22  (−1.25, 1.69) | 0.11  (−1.42, 1.64) | | 0.74 | 0.72 |
| Model 3‡ | 0 | 0.77  (−0.68, 2.22) | 0.29  (−1.18, 1.76) | 0.42  (−1.13, 1.97) | | 0.77 | 0.91 |
|  | Stroop Test | | | | | | |
| Model 1* | 0 | −0.05  (−1.30,1.20) | 0.04  (−1.21,1.29) | 0.19  (−1.12, 1.50) | | 0.99 | 0.38 |
| Model 2┼ | 0 | −0.23  (−1.46, 1.00) | −0.27  (−1.52, 0.98) | −0.11 (−1.42,1.20) | | 0.97 | 0.64 |
| Model 3‡ | 0 | −0.25  (−1.48, 0.98) | −0.48  (−1.73, 0.77) | −0.62  (−1.93, 0.69) | | 0.81 | 0.72 |
|  |  |  |  |  | |  |  |
|  | Quartiles of E-Selectin (ng/ml) | | | | | P value  (3 df) | P trend |
|  | Q1 (520) | Q2 (521) | Q3 (521) | Q4 (521) | |  |  |
|  | 3.45—21.81 | 21.82—31.01 | 31.02—42.54 | 42.55—86.21 | |  |  |
|  | Rey Auditory Verbal Learning Test (RAVLT) | | | | | | |
| Model 1* | 0 | 0.36  (0.01, 0.71) | −0.08  (−0.43, 0.27) | 0.02  (−0.33, 0.37) | | 0.06 | 0.31 |
| Model 2┼ | 0 | 0.37 (0.02,0.72) | −0.07 (−0.42,0.28) | 0.05 (−0.30,0.40) | | 0.06 | 0.39 |
| Model 3‡ | 0 | 0.37  (0.02, 0.72) | −0.06  (−0.41, 0.29) | 0.10  (−0.27, 0.47) | | 0.06 | 0.52 |
|  | Digit Symbol Substitution Test | | | | | | |
| Model 1* | 0 | −0.07  (−1.69, 1.56) | −0.78  (−2.43, 0.87) | −1.95  (−3.66, −0.24) | | 0.09 | <0.01 |
| Model 2┼ | 0 | −0.06 (−1.69,1.57) | −0.73 (−2.38,0.92) | −1.76 (−3.47,−0.05) | | 0.15 | 0.02 |
| Model 3‡ | 0 | 0.00  (−1.63, 1.63) | −0.51  (−2.18, 1.16) | −1.27  (−3.05, 0.51) | | 0.45 | 0.09 |
|  | Stroop Test | | | | | | |
| Model 1* | 0 | 0.14  (−1.23, 1.51) | 0.99  (−0.40, 2.38) | 0.52  (−0.91, 1.95) | | 0.51 | 0.31 |
| Model 2┼ | 0 | 0.13 (−1.24,1.50) | 0.93 (−0.46,2.32) | 0.40 (−1.03,1.83) | | 0.56 | 0.43 |
| Model 3‡ | 0 | 0.01  (−0.13, 0.15) | 0.63  (−0.78, 2.04) | −0.16  (−0.91, 1.33) | | 0.68 | 0.96 |
|  |  | | | | |  |  |
|  | Quartiles of Fractalkine (ng/ml) | | | | | P value (3df) | P trend |
|  | Q1 (N=617) | Q2 (N=617) | Q3 (N=617) | Q4 (N=617) | |  |  |
|  | 0.00-0.45 | 0.46-0.51 | 0.52-0.61 | 0.62-13.12 | |  |  |
|  | Rey Auditory Verbal Learning Test (RAVLT) | | | | | | |
| Model 1* | 0 | −0.06  (−0.37, 0.25) | −0.24  (−0.55,0.07) | −0.20  (−0.51,0.11) | | 0.42 | 0.20 |
| Model 2┼ | 0 | −0.08 (−0.39, 0.23) | −0.24 (−0.55, 0.07) | −0.21 (−0.52, 0.10) | | 0.44 | 0.21 |
| Model 3‡ | 0 | −0.08  (−0.39,0.23) | −0.23  (−0.54,0.08) | −0.17  (−0.48,0.14) | | 0.51 | 0.29 |
|  | Digit Symbol Substitution Test (DSST) | | | | | | |
| Model 1* | 0 | 0.19  (−1.32,1.70) | 0.10  (−1.42,1.63) | 0.11  (−1.44, 1.66) | | 1.00 | 0.95 |
| Model 2┼ | 0 | 0.07  (0.01, 0.12) | 0.02  (−1.48, 1.53) | −0.02  (−1.55, 1.51) | | 1.00 | 0.90 |
| Model 3‡ | 0 | 0.08  (−1.43, 1.59) | 0.00  (−1.51, 1.51) | −0.09  (−1.62, 1.44) | | 1.00 | 0.92 |
|  | Stroop Test | | | | | | |
| Model 1* | 0 | 0.61  (−0.69,1.90) | 0.78  (−0.53,20.9) | 0.27  (−1.04, 1.58) | 0.65 | | 0.69 |
| Model 2┼ | 0 | 0.71  (−0.58, 2.00) | 0.81  (−0.48, 2.10) | 0.32 (−0.99,1.63) | 0.59 | | 0.81 |
| Model 3‡ | 0 | 0.69  (−0.60, 1.98) | 0.88  (−0.41, 2.17) | 0.44  (−0.87, 1.75) | 0.57 | | 0.89 |

Each regression coefficient is the mean cognitive test score difference from the cognitive test score inquartile 1 of the given cellular adhesion molecule (CAM). P value (3 df) is based on an F-test for any difference in cognitive score means among the 4 CAM quartiles. P trend is computed across the continuous CAM variable.

*Model 1: adjusted for age, race, sex, education, and center

†Model 2: model 1+ smoking, alcohol, physical activity, and a priori diet quality score

‡Model 3: model 2+ body mass index, elevated blood pressure (including use of antihypertensive medication), diabetes, blood lipids, and C-reactive protein.

**Online Table 2.** Regression coefficients (95%CI) of year 25 cognitive test scores across quartiles of year 15 VCAM-1, P selectin, and Fractalkine. (N=2,775 for VCAM-1, N= 2,788 for P-selectin, N=2,645 and N=2,578 for fractalkine)

|  | Quartiles of VCAM-1  (ng/ml) | | | | | | | P value (3df) | | P trend |
| --- | --- | --- | --- | --- | --- | --- | --- | --- | --- | --- |
|  | Q1 (N=693) | Q2 (N=694) | | Q3 (N=694) | | Q4 (N=694) | |  | |  |
|  | 167.22—418.37 | 418.38—506.52 | | 506.53—606.79 | | 606.80—2279.61 | |  | |  |
|  | Rey Auditory Verbal Learning Test (RAVLT) | | | | | | | | | |
| Model 1* | 0 | 0.16  (−0.13, 0.45) | | 0.08  (−0.23, 0.39) | | 0.04  (−0.27, 0.35) | | 0.74 | | 0.57 |
| Model 2┼ | 0 | 0.15 (−0.14,0.44) | | 0.07 (−0.24,0.38) | | 0.02 (−0.29,0.33) | | 0.75 | | 0.66 |
| Model 3‡ | 0 | 0.14  (−0.15, 0.43) | | 0.06  (−0.25, 0.37) | | 0.01  (−0.32, 0.34) | | 0.77 | | 0.63 |
|  | Digit Symbol Substitution Test (DSST) | | | | | | | | | |
| Model 1* | 0 | 0.14  (−1.27, 1.55) | | −1.05  (−2.50, 0.40) | | 0.12  (−1.34, 1.63) | | 0.29 | 0.25 | |
| Model 2┼ | 0 | −0.06 (−1.47,1.35) | | −1.34 (−2.77,0.09) | | −0.27 (−1.76,1.22) | | 0.21 | 0.12 | |
| Model 3‡ | 0 | −0.13  (−1.54, 1.28) | | −1.36  (−2.81, 0.09) | | −0.30  (−1.21, 1.81) | | 0.22 | 0.17 | |
|  | Stroop Test | | | | | | | | | |
| Model 1* | 0 | −0.23  (−0.99, 1.45) | | −0.15  (−1.38, 1.08) | | −1.01  (−2.28, 0.26) | | 0.39 | 0.47 | |
| Model 2┼ | 0 | −0.15 (−1.37,1.07) | | −0.03 (−1.26,1.20) | | −0.84 (−2.11,0.43) | | 0.50 | 0.61 | |
| Model 3‡ | 0 | −0.07  (−1.29, 1.14) | | 0.04  (−1.19,1.27) | | −0.70  (−1.97, 0.57) | | 0.62 | 0.63 | |
|  |  |  | |  | |  | |  |  | |
|  | Quartiles of P-selectin (ng/ml) | | | | | | | P value (3df) | P trend | |
|  | Q1 (N=697) | Q2 (N=697) | | Q3 (N=697) | | Q4 (N=697) | |  |  | |
|  | 0.42-29.96 | 29.97-35.62 | | 35.63-41.95 | | 41.96-160.19 | |  |  | |
|  | Rey Auditory Verbal Learning Test (RAVLT) | | | | | | | | | |
| Model 1* | 0 | 0.02  (−0.96, 1.00) | | −0.02  (−0.33, 0.29) | | −0.05 (−0.36, 0.26) | | 0.97 | 0.96 | |
| Model 2┼ | 0 | 0.02 (−0.27,0.31) | | −0.02  (−0.33, 0.29) | | −0.04 (−0.35,0.27) | | 0.98 | 0.83 | |
| Model 3‡ | 0 | 0.03  (−0.26, 0.32) | | 0.02  (−0.29, 0.33) | | 0.02  (−0.29, 0.33) | | 1.00 | 0.60 | |
|  | Digit Symbol Substitution Test (DSST) | | | | | | | | | |
| Model 1* | 0 | 0.83  (−0.58, 2.24) | | 0.49  (−0.94, 1.92) | | −0.24  (−1.71, 1.23) | | 0.44 | 0.42 | |
| Model 2┼ | 0 | 0.95 (−0.46,2.36) | | 0.70 (−0.73,2.13) | | 0.17 (−1.30,1.64) | | 0.51 | 0.87 | |
| Model 3‡ | 0 | 1.10  (−0.31, 2.51) | | 0.98  (−0.45, 2.41) | | 0.53  (−0.96, 2.02) | | 0.40 | 0.66 | |
|  | Stroop Test | | | | | | | | | |
| Model 1* | 0 | −0.15  (−1.37, 1.06) | | −0.43  (−1.66, 0.80) | | −0.40  (−1.65, 0.85) | | 0.90 | 0.57 | |
| Model 2┼ | 0 | −0.22 (−1.43,1.00) | | −0.54 (−1.77,0.69) | | −0.60 (−1.87,0.67) | | 0.77 | 0.36 | |
| Model 3‡ | 0 | −0.37  (−1.59, 0.85) | | −0.85  (−2.08, 0.38) | | −1.03  (−2.30, 0.24) | | 0.39 | 0.08 | |
|  |  |  | |  | |  | |  |  | |
|  | Quartiles of E-Selectin (ng/ml) | | | | | | | P value  (3 df) | P trend | |
|  | Q1 (661) | Q2 (661) | | Q3 (662) | | Q4 (661) | |  |  | |
|  | 5.17—24.64 | 24.65—32.97 | | 32.98—42.48 | | 42.49—85.07 | |  |  | |
|  | Rey Auditory Verbal Learning Test (RAVLT) | | | | | | | | | |
| Model 1* | 0 | −0.05  (−0.36, 0.26) | | −0.11  (−0.42, 0.20) | | −0.06  (−0.39, 0.27) | | 0.92 | 0.79 | |
| Model 2┼ | 0 | −0.04  (−0.35, 0.27) | | −0.11  (−0.42, 0.20) | | −0.07  (−0.40, 0.26) | | 0.93 | 0.75 | |
| Model 3‡ | 0 | −0.02  (−0.33, 0.29) | | −0.07  (−0.38, 0.24) | | −0.02  (−0.35, 0.31) | | 0.97 | 0.81 | |
|  | Digit Symbol Substitution Test (DSST) | | | | | | | | | |
| Model 1* | 0 | 0.09  (−1.36, 1.54) | | −1.37  (−2.84, 0.18) | | −1.83  (−3.33, −0.32) | | 0.02 | <0.01 | |
| Model 2┼ | 0 | 0.09  (−1.34, 1.52) | | −1.29  (−2.74, 0.16) | | −1.56  (−3.07, −0.05) | | 0.06 | <0.01 | |
| Model 3‡ | 0 | 0.28  (−1.15, 1.71) | | −0.96  (−2.43, 0.51) | | −1.07  (−2.64, 0.50) | | 0.22 | 0.07 | |
|  | Stroop Test | | | | | | | | | |
| Model 1* | 0 | 0.23  (−1.00, 1.46) | | 0.95  (−0.30, 2.20) | | 0.70  (−0.59, 1.99) | | 0.45 | 0.16 | |
| Model 2┼ | 0 | 0.23  (−1.00, 1.46) | | 0.92  (−0.31, 2.15) | | 0.59  (−0.70, 1.88) | | 0.50 | 0.21 | |
| Model 3‡ | 0 | 0.08  (−1.15, 1.31) | | 0.56  (−0.69, 1.81) | | −0.01  (−1.36, 1.34) | | 0.76 | 0.92 | |
|  |  |  | |  | |  | |  |  | |
|  | Quartiles of Fractalkine (ng/ml) | | | | | | | P value (3df) | P trend | |
|  | Q1 (N=644) | | Q2 (N=645) | | Q3 (N=645) | | Q4 (N=644) |  |  | |
|  | 0.005-0.48 | | 0.49-0.56 | | 0.57-0.64 | | 0.65-3.42 |  |  | |
|  | Rey Auditory Verbal Learning Test (RAVLT) | | | | | | | | | |
| Model 1* | 0 | | −0.03  (−0.34, 0.28) | | −0.08  (-0.38, 0.24) | | −0.11  (−0.42, 0.20) | 0.91 | 0.46 | |
| Model 2┼ | 0 | | −0.04 (−0.35,0.27) | | −0.08 (−0.39,0.23) | | −0.11 (−0.42,0.20) | 0.91 | 0.46 | |
| Model 3‡ | 0 | | −0.02  (−0.33, 0.29) | | −0.07  (−0.38, 0.24) | | −0.09  (−0.40, 0.22) | 0.93 | 0.55 | |
|  | Digit Symbol Substitution Test (DSST) | | | | | | | | | |
| Model 1* | 0 | | 1.31  (−0.16, 2.78) | | 1.26  (−0.21,2.73) | | 0.25  (−1.22, 1.72) | 0.17 | 0.14 | |
| Model 2┼ | 0 | | 1.11 (−0.36,2.58) | | 1.20 (−0.27,2.67) | | 0.03 (−1.44,1.50) | 0.19 | 0.09 | |
| Model 3‡ | 0 | | 1.13  (−0.32, 2.58) | | 1.18  (−0.27, 2.63) | | 0.04  (−1.43, 1.51) | 0.19 | 0.09 | |
|  | Stroop Test | | | | | | | | | |
| Model 1* | 0 | | 0.68  (−0.57, 1.93) | | 0.56  (−0.69,1.81) | | 0.33  (−0.92, 1.58) | 0.73 | 0.55 | |
| Model 2┼ | 0 | | 0.75 (−0.50,2.00) | | 0.59 (−0.66,1.84) | | 0.42 (−0.83,1.67) | 0.67 | 0.48 | |
| Model 3‡ | 0 | | 0.72  (−0.53, 1.97) | | 0.59  (−0.66, 1.84) | | 0.43  (−0.82, 1.68) | 0.69 | 0.53 | |

Each regression coefficient is the mean cognitive test score difference from the cognitive test score in quartile 1 of the given cellular adhesion molecule (CAM). P value (3 df) is based on an F-test for any difference in cognitive score means among the 4 CAM quartiles. P trend is computed across the continuous CAM variable.

*Model 1: adjusted for age, race, sex, education, and center

†Model2: model 1+ smoking, alcohol, physical activity, a priori diet quality score

‡Model 3: model 2+ body mass index, elevated blood pressure (including use of antihypertensive medication), diabetes, blood lipids, C-reactive protein.
